# Enhanced Transgene Expression by Optimization of Poly A in Transfected CHO Cells

**DOI:** 10.3389/fbioe.2022.722722

**Published:** 2022-01-24

**Authors:** Xiao-yin Wang, Qiu-jie Du, Wei-li Zhang, Dan-hua Xu, Xi Zhang, Yan-long Jia, Tian-yun Wang

**Affiliations:** ^1^ School of Basic Medical Sciences, Xinxiang Medical University, Xinxiang, China; ^2^ College of Pharmacy, Xinxiang Medical University, Xinxiang, China

**Keywords:** Chinese hamster ovary cells, poly A elements, transgene expression, stability, optimization

## Abstract

The generation of the stable, high-level recombinant protein-producing cell lines remains a significant challenge in the biopharmaceutical industry. Expression vector optimization is an effective strategy to increase transgene expression levels and stability, and the choice of suitable poly A element is crucial for the expression of recombinant protein. In this study, we investigated the effects of different poly A elements on transgene expression in Chinese hamster ovary (CHO) cells. Five poly A elements, including bovine growth hormone (BGH), mutant BGH, herpes simplex virus type 1 thymidine kinase (HSV-TK), SV40, and a synthetic (Synt) poly A, were cloned into the expression vector and transfected into CHO cells. The results indicated the SV40 and Synt poly A sequences can significant improve *eGFP* transgene expression in stable transfected CHO cells and maintain long-term expression. However, qPCR results showed that the *eGFP* expression at protein level was not related to the gene copy number and mRNA level. Importantly, the SV40 and Synt poly A elements decreased the variation of *eGFP* transgene expression. Furthermore, it also showed that the SV40 and Synt poly A elements induced higher levels of adalimumab expression. In conclusion, SV40 poly A and Synt poly A are stronger elements that increase stable transgene expression and decrease the variation of expression, and the choice of suitable poly A element is helpful to improve the expression of recombinant protein.

## Introduction

Chinese hamster ovary (CHO) cells are the most frequently used expression system for the production of recombinant therapeutic proteins (Tripathi and Shrivastava 2019; Kuo et al., 2018). However, the main bottlenecks during recombinant protein production are low levels, variability of transgene expression, and unstable of expression level during long-time culture (Santilli et al., 2011; Jazayeri et al., 2018). To achieve stable, high recombinant protein production, cell line engineering, growth medium modification, and the incorporation of *cis*-acting elements were performed, and obtained the ideal purpose (Jia et al., 2018; Guo et al., 2020). Among these approaches, expression vector optimization is an effective strategy to increase transgene expression level and stability.

The expression of a heterologous gene in host cells depends on some components in the expression vector, such as an enhancer, promoter, intron, poly A, integration site, and regulatory sequences. Therefore, the proper selection of these elements can increase recombinant protein expression levels and stability (Lee JS et al., 2018; Lee CP et al., 2018; Yeo et al., 2017). However, most studies have focused on the role of the enhancer, internal ribosome entry site (IRES), intron, promoters, and combinations of regulatory elements with promoters (Hunter et al., 2019; Skipper et al., 2019; Yeo et al., 2017; Chai et al., 2018), few studies have been performed on the effects of poly A elements on transgene expression in CHO cells.

The promoter element is essential for mediating the initiation of transcription, and in its location at the other end of the reading frame, the poly A element affects the stability of RNA products and plasmid DNA susceptible to nuclease attack (Ribeiro et al., 2004; Ptitsyna et al., 2017; Hamilton et al., 2019). There are several polyA elements related to transgene expression in mammalian animal cell system, polyA from the bovine growth hormone (BGH) gene can mediate efficient transcript termination and polyadenylation to heterologous genes (Pfarr et al., 1986; Goodwin and Rottman 1992) and the SV40 and the synthetic poly A elements present significant improvements in nuclease resistance (Azzoni et al., 2007), the synthetic poly A is based on the highly efficient poly A signal of the rabbit beta-globin gene, and the highest number of plasmid copies was obtained with plasmid contains Synt poly A in transfected HeLa cells (Levitt et al., 1989). And systematic research of effect of poly A on transgene in CHO cells was not reported. In this study, we investigated the effects of five poly A elements on transgene expression in transfected CHO cells.

## Materials and Methods

### Vector Construction

**FIGURE 1:**
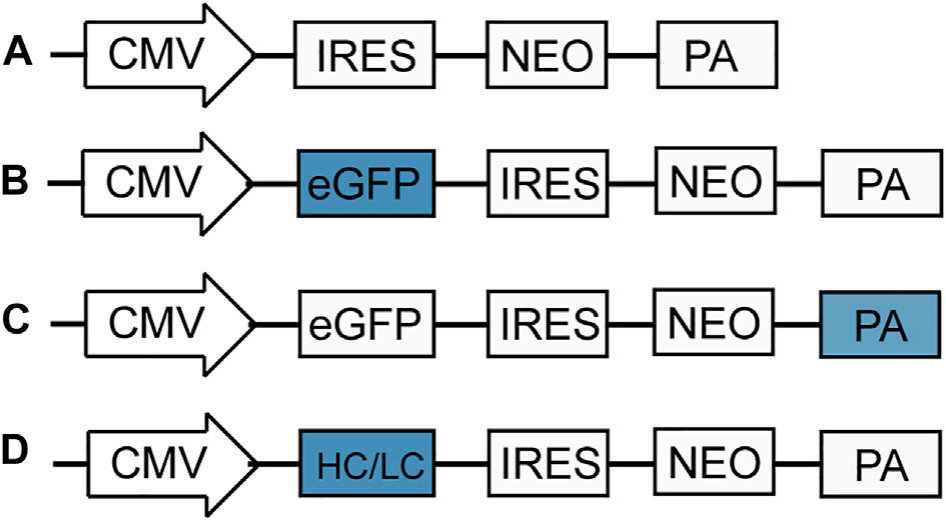
Schematic representation of vectors used in this study. **(A)** pIRES-neo expression vector that contains CMV promoter, internal ribosome entry site (IRES), neomycin (neo) and BGH poly A; **(B)** *eGFP* gene was cloned downstream CMV promoter of pIRES-neo vector to produce the pIRES-EGFP vector; **(C)** Four different poly A elements, including M BGH, Synt, HSV TK and SV40 poly A replaced the BGH poly A of pIRES-EGFP vector by fusion cloning technique to produce the vectors containing different poly A; **(D)** A dalimumab heavy chain (HC) and light chain (LC) gene replaced the *eGFP* of pIRES-GFP-poly A vector to produce the vectors containing adalimumab.

Based on the previously reported sequences (Denome and Cole, 1988 Levitt et al., 1989; Azzoni et al., 2007), five different poly A elements were studied, mutant bovine growth hormone polyadenylation signal (M BGH poly A), a synthetic polyadenylation sequence (Synt poly A), herpes simplex virus type 1 (HSV) thymidine kinase polyadenylation signal (HSV-TK poly A) and simian virus 40 late polyadenylation signal (SV40 poly A) were artificially synthesized by General Biosystems (Chuzhou, China). The new synthetic poly A elements replaced the original bovine growth hormone polyadenylation signal (BGH poly A) of the pIRES-EGFP vector (Figure 1B) (Chen et al., 2017), that was constructed based on the pIRES-neo vector (Figure 1A), The information and sequence of pIRES-eGFP was provided in Supplementary Figure S1. The new plasmids were named as pIRES-EGFP-M BGH, pIRES-EGFP-Synt, pIRES-EGFP-HSV-TK, and pIRES-EGFP-SV40, respectively (Figure 1C).

To further explore the role of the selected poly A elements on recombinant protein production, expression vectors containing adalimumab were constructed according to standard molecular methods. Briefly, the adalimumab heavy chain (HC) and light chain (LC) genes were artificially synthesized and used to replace the *eGFP* gene in the pIRES-EGFP-poly A vectors (Figure 1D). The sequence of five ploy A elements, adalimumab heavy chain and light chain were provided in Supplementary Figure S2.

### Cell Culture and Transfection

CHO-S cells (Life Technologies # A11557-01) were maintained in Dulbecco’s modified Eagle’s medium (DMEM)/F12 medium (ProteinEasy Biological Products Co., Ltd., Xinxiang, China) supplemented with 1% penicillin-streptomycin solution (ProteinEasy Biological Products Co., Ltd., Xinxiang, China) and 10% fetal bovine serum (Gibco, Carlsbad, CA), in a 37°C humidified incubator with 5% CO_2_. For transfection, CHO-S cells were counted and inoculated into 12-well plates at approximately 4 × 10^5^ cells/well. After reaching 80–90% confluence, cells were transfected with the vectors described above using Lipofectamine 2000 reagent (Invitrogen, Carlsbad, CA, USA) following the manufacturer’s instructions. 4 µl Lipofectamine 2000 transfection reagent per 2 µg plasmid DNA was transfected into each well, all experiments were repeated three times.

### Transient Expression

At 48 h post-transfection, eGFP transient expression was observed by fluorescence microscopy (Nikon ECLIPSE, Nikon, Japan) and mean fluorescence intensity (MFI) was detected by flow cytometry as previously described (Wang et al., 2019). Briefly, cells were collected after pancreatic enzyme digestion, a total of 100,000 fluorescent events were acquired using a 530/15 bandpass filter for the green fluorescent signal, which was acquired with an emission wave length of 530 nm. Data acquisition and analyses were performed using Flow Jo software 7.6 (Tree Star, Ashland, OR).

### Stable and Long-Term Stable Expression

The stable transfected cell pools were selected using 800 μg/ml G418 at 48 h post-transfection. The non-transfected cells were eliminated after approximately 7–10 days, then the cells were cultured in medium containing 500 μg/ml G418. At 30 days after transfection, when the cell confluence reached 90%, cells transfected with plasmids containing the different poly A elements were collected, and eGFP stable expression was measured using flow cytometry.

For the analysis of long-term stability, the stable cell lines were further cultured in G418-containing medium (500 μg/ml) for 120 days. During this period, the medium was changed according to the growth of the cells, and the cells were routinely sub-cultured every 2–3 days. At 120 days after transfection, cells were collected and the eGFP expression level was analyzed by flow cytometry.

### Real-Time Quantitative PCR

*eGFP* relative copy numbers and mRNA expression levels were determined using stable transfected cells at 120 days after transfection. Genomic DNA was isolated using a genomic DNA extraction kit (ProteinEasy Biological Products Co., Ltd., Xinxiang, China). Total RNA was isolated using the RNA pure Tissue Kit (Beijing Com Win Biotech Co., Ltd., Beijing, China), then was converted to cDNA using a cDNA Reverse Transcription Kit (Beyotime Biotech, Shanghai, China). The primers used for qPCR reaction were as follows: *eGFP*, 5′-GCT​GGT​TTA​GTG​AAC​CGT​CAG-3′ and 5′-AGG​TGG​CAT​CGC​CCT​CGC​CC-3′; glyceraldehyde phosphate dehydrogenase (GAPDH) (internal reference), 5′-CGA​CCC​CTT​CAT​TGA​CCT​C-3′ and 5′-CTC​CAC​GAC​ATA​CTC​AGC​ACC-3′. Genomic DNA and cDNA were used for qPCR using the SYBR Premix Ex Taq (Takara Bio, Beijing, China) with ABI 7500 SYBR fluorescence quantitative PCR instrument (Applied Biosystems, Foster, CA, USA), and the results were analyzed by 7,500 Fast System SDS Software. The reaction mixture (10 μL) consisted of 5 μL SYBR Green (TAKARA, Dalian, China), 4 μL template DNA (0.05 μg/μL), 0.3 μL each of the forward and reverse primers (10 nmol/ml), and 0.4 μL deionized water. The PCR protocol was as follows: 95°C for 3 min, 40 cycles of 95°C for 10 s, followed by 60°C for 30 s, 95°C for 15 s, 60°C for 60 s. Through qPCR, the Ct value was obtained. All samples were evaluated three times, and relative *eGFP* copy numbers and the level of *eGFP* mRNA was calculated using the 2^−ΔΔCt^ method (Livak and Schmittgen, 2001).

### Analysis of Adalimumab Expression

After co-transfection with the adalimumab HC and LC constructs, cells were selected in the presence of G418 as described above to establish stable CHO cell colonies. Then, the cells were suspended in a serum-free medium (ProteinEasy Biological Products Co., Ltd., Xinxiang, China) without antibiotic at a concentration of 5 × 10^6^ cells/mL with a working volume of 30 ml in 125-ml Corning shake flasks. After 7 days, supernatants were collected, and adalimumab expression was analyzed by Western blotting and enzyme-linked immunosorbent assay (ELISA) as previously described (Wang et al., 2018). For Western blot analysis, 20 μl aliquots of the supernatant containing adalimumab were electrophoresed on a 10% SDS-PAGE gel, after which proteins were transferred to polyvinylidene fluoride membranes and incubated with 1:5,000 dilutions of goat anti-human antibody (EarthOx, San Francisco, CA, USA) for 2 h at room temperature. After washing with PBST (PBS with 0.1% Tween20), the protein bands were visualized using an enhanced chemiluminescence substrate kit (Beyotime Biotech Co., Ltd., Shanghai, China). At the same time, adalimumab expression was quantified with ELISA (ELK Biotechnology Co., Ltd., Wuhan, China) according to the provided protocol. Cell concentration was determined with a Countstar cell counter, and adalimumab specific productivity (pg/cell/day) was calculated.

### Statistical Analysis

All statistical analyses were carried out using the SPSS 22.0 software (SPSS Inc., Chicago, IL, United States). Data were analyzed using independent sample t-tests and are reported as mean ± standard deviation. *p* value less than 0.05 was considered statistically significant.

## Results

### Evaluation the Effect of Different Poly A on eGFP Transient Expression

**FIGURE 2:**
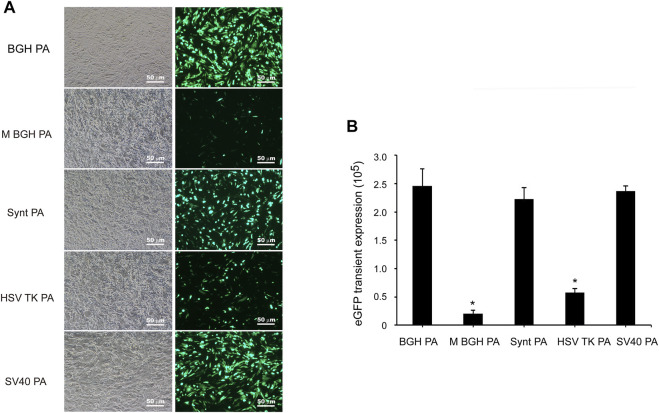
Effect of different poly A containing vector on transient transgene expression. **(A)** Vectors containing different poly A were transfected into CHO cells at 48 post-transfection, fluorescence intensity was observed under fluorescence microscopy. **(B)** The cells were collected, and the mean fluorescence intensity was measured by flow Cytometry, results are expressed as the mean values obtained from three independent experiments, and SEM is indicated (*p < 0.05).

The vectors containing different poly A sequences were constructed and transfected into CHO cells. At 48 h post-transfection, the mean fluorescence intensity (MFI) of eGFP was observed using fluorescence microscopy and measured by flow cytometry. As shown in Figure 2, the distinct differences in the transient expression of eGFP appeared between the different poly A-containing vectors. Transient eGFP expression with the BGH poly A-containing vector (i.e., control vector) was higher than that with the other vectors, suggesting that the M BGH, Synt, HSV-TK, and SV40 poly A sequences did not increase the transient expression of the recombinant protein. Statistical analysis showed no difference in the transient eGFP expression between vectors with Synt, SV40, and BGH poly A, and the transient eGFP expression from the vectors with the M BGH and HSV-TK poly A was significantly lower than that of the BGH poly A (Figure 2, *p* < 0.05), while the M BGH poly A vector showed the lowest transient expression (12.2-fold decrease).

### Synt Poly A and SV40 Poly A Enhance Stable Transgene Expression

**FIGURE 3:**
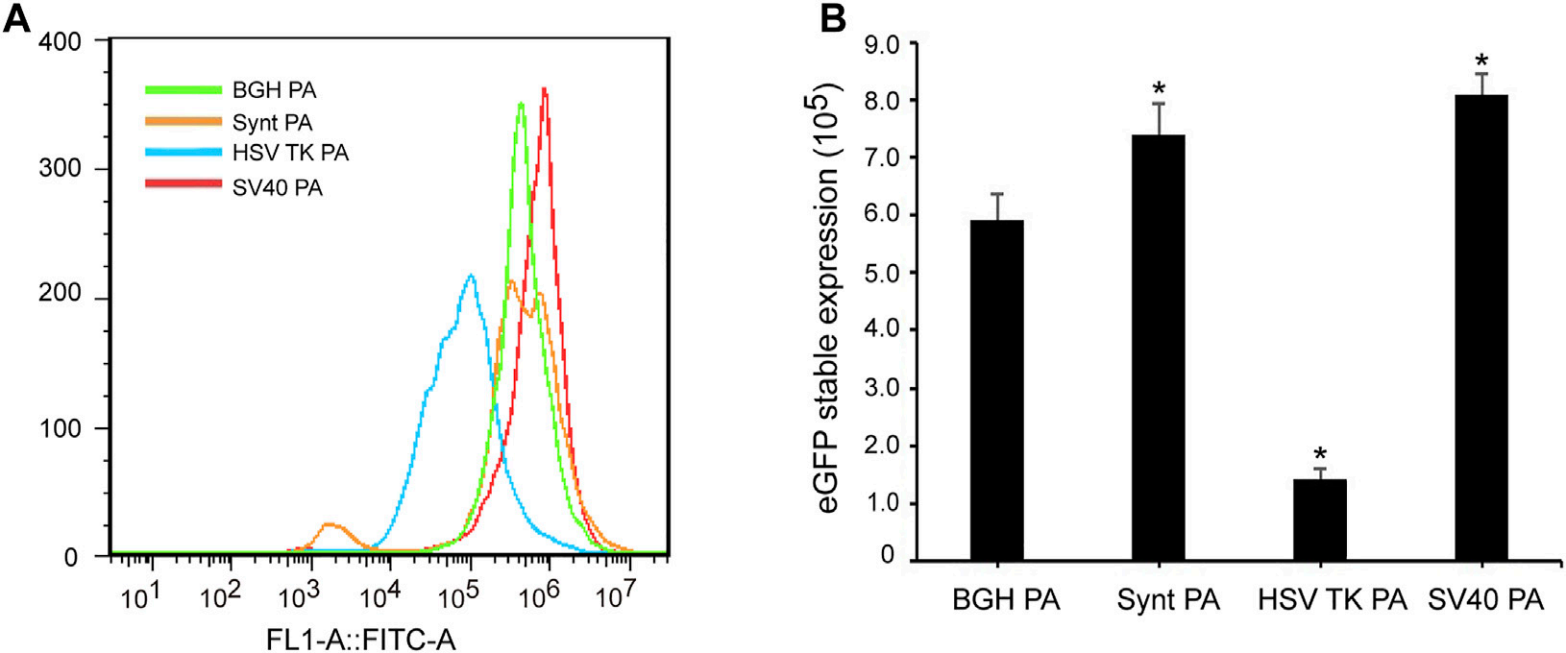
Effect of different poly A containing vector on stable transgene expression. Cells were collected under G418 screening at 30 days post-transfection, recombinant protein expression was tested using flow cytometric analysis. **(A)** The mean fluorescence intensity was measured by flow cytometry; **(B)** Analysis of eGFP stable expression. Error bars indicate standard deviation between triplicates. Compared with BGH poly A, *p* < 0.05.

To analyze the stability of the poly A elements, cells transfected with the different vectors were further cultured under G418 selection to generate stable cell lines. At day 30 post-transfection, the cells were collected, and fluorescence intensity was detected by flow cytometry (Figure 3A). The MFI of cells transfected with the HSV-TK poly A vector was lower than that of the BGH poly A, while cells transfected with the Synt poly A and SV40 poly A containing vectors exhibited higher MFI than that of BGH poly A (Figure 3B). The eGFP expression level of the BGH poly A vector was set as 1.0, the values of the Synt poly A and SV40 poly A sequences were 1.24 and 1.37, respectively, suggesting that both of these poly A elements were able to enhance transgene expression in stable transfected CHO cells. However, no eGFP expression was detected in cells transfected with M BGH poly A at 30 days post-transfection.

### Synt Poly A and SV40 Poly A Enhance Long-Term Stability and Reduce Variation of Expression

**FIGURE 4:**
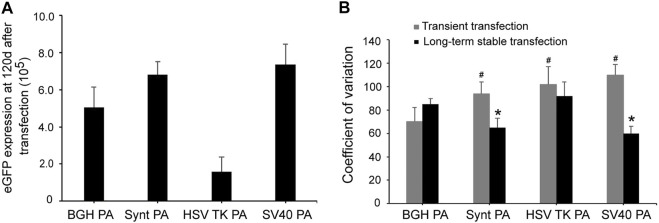
**(A)** Long-term eGFP expression stability in transfected CHO cells. Cells were collected, and FACSCalibur estimated the eGFP fluorescence profile at 120 days after transfection. **(B)** The CV of transient expression and long-term stable expression of eGFP. CV values are expected to reflect variations in transgene expression. The gray bar represents CV of temporary transfection cells of eGFP that were tested at 48 h post-transfection. The black bar represents CV of stable transfection cells of eGFP at 120 days after transfection. The results are the mean values obtained for three independent experiments, and standard deviation is indicated. Compared with BGH poly A, *p* < 0.05.

For long-term stability analysis, CHO cells were passaged for 120 days after transfection and collected to test eGFP expression by flow cytometry. Consistent with the results from the stable expression analysis, SV40 poly A displayed the highest eGFP expression, followed by the Synt poly A (Figure 4A). Compared with the BGH poly A, the SV40 and Synt poly A vectors exhibited higher eGFP expression by 1.45- and 1.32-fold, respectively. In contrast, the HSV-TK poly A did not increase eGFP expression levels at day 120 post-transfection.

The coefficient of variation (CV) reflects the variety of expression levels. In transient expression, the CV for the vectors containing Synt, HSV-TK, and SV40 poly A sequences were higher than that of the BGH poly A (*p* < 0.05, Figure 4B). However, in long-term stable transfected cells, the CV of HSV-TK poly A was still higher than that of BGH poly A, whereas that of Synt and SV40 poly A were lower (*p* < 0.05, Figure 4B), suggesting that the Synt and SV40 poly A elements can decrease the variation of transgene expression in long-term stable transfected cells.

### Transgene Expression Level is Not Related to the Copy Number and mRNA Level

**FIGURE 5:**
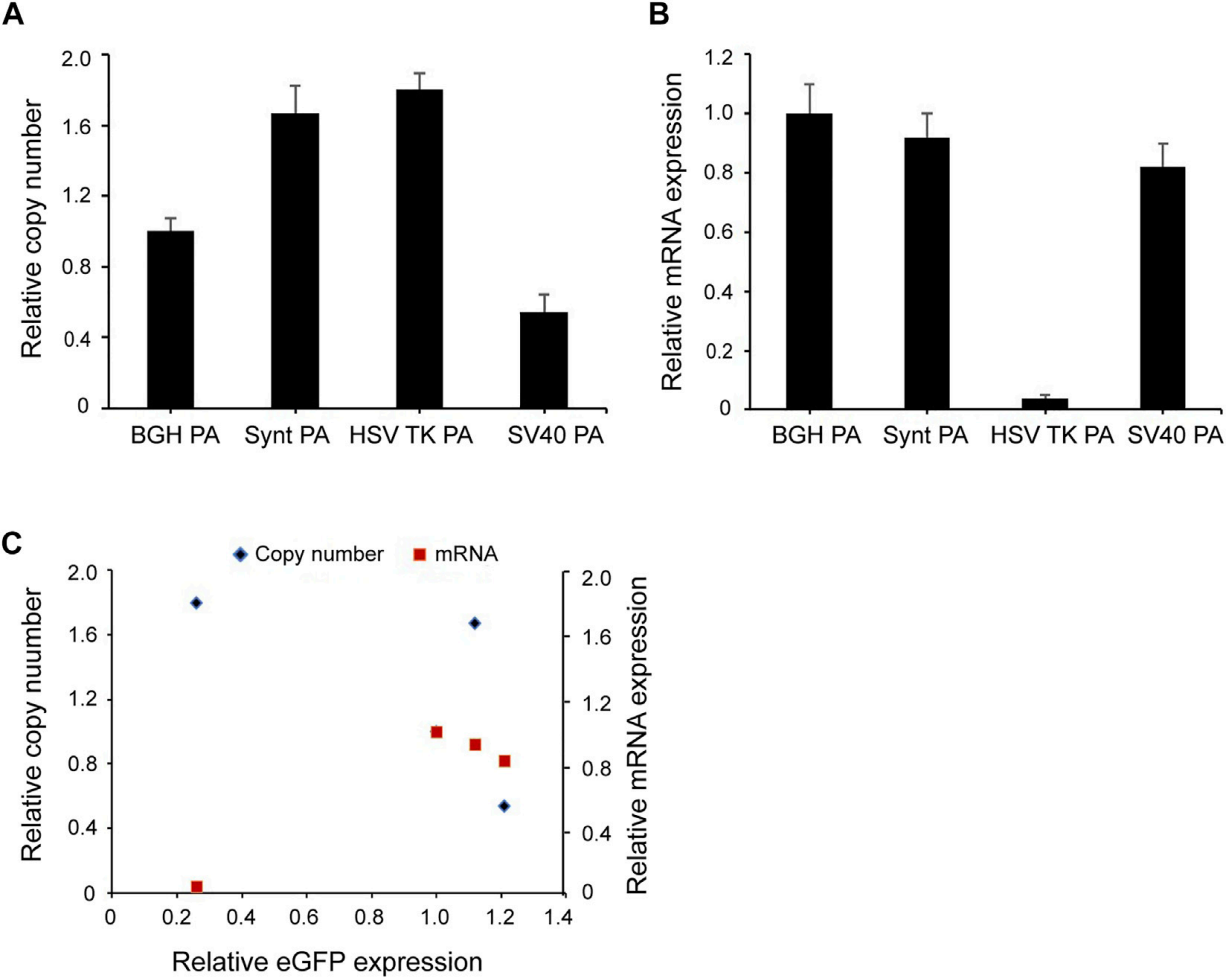
**(A,B)** Relative *eGFP* copy number and mRNA expression level in long-term stable transfected cells. Fluorescent quantitative PCR was used to analyze *eGF*P gene copy numbers and mRNA expression. Relative copy numbers and mRNA expression levels were calculated using the 2^−ΔΔCt^ method and were normalized to the BGH poly A vector whose value was set to 1. **(C)** Correlation analysis between the relative *eGFP* copy (blue diamond), mRNA expression (red square) and relative eGFP expression in long-term stable transfected CHO cells. Results were obtained from three independent experiments. The standard error of the mean (S.E.M.) was indicated.

To investigate whether the Synt poly A and SV40 poly A elements influence transgene expression by increasing the copy number and mRNA level of the integrated transgene, qPCR was carried out. The data were normalized to the BGH poly A vector, and the results indicated that the highest expression of the SV40 poly A showed the lowest copy number, and that of the HSK TV poly A (with a relatively lower expression) possessed the highest copy number. These results suggest that the *eGFP* expression level was not related to the gene copy number in the long-term stable transfected cells (Figures 5A,C).

For mRNA level, the BGH poly A vector yielded the highest mRNA expression levels, followed by the Synt poly A, however, the highest expressing vector (the SV40 poly A containing construct) did not exhibit highest mRNA expression levels (Figure 5B). The results from the correlation analysis indicated that there was no correlation between eGFP expression and mRNA level (Figure 5C).

### Synt Poly A and SV40 Poly A Enhance Adalimumab Expression

**FIGURE 6:**
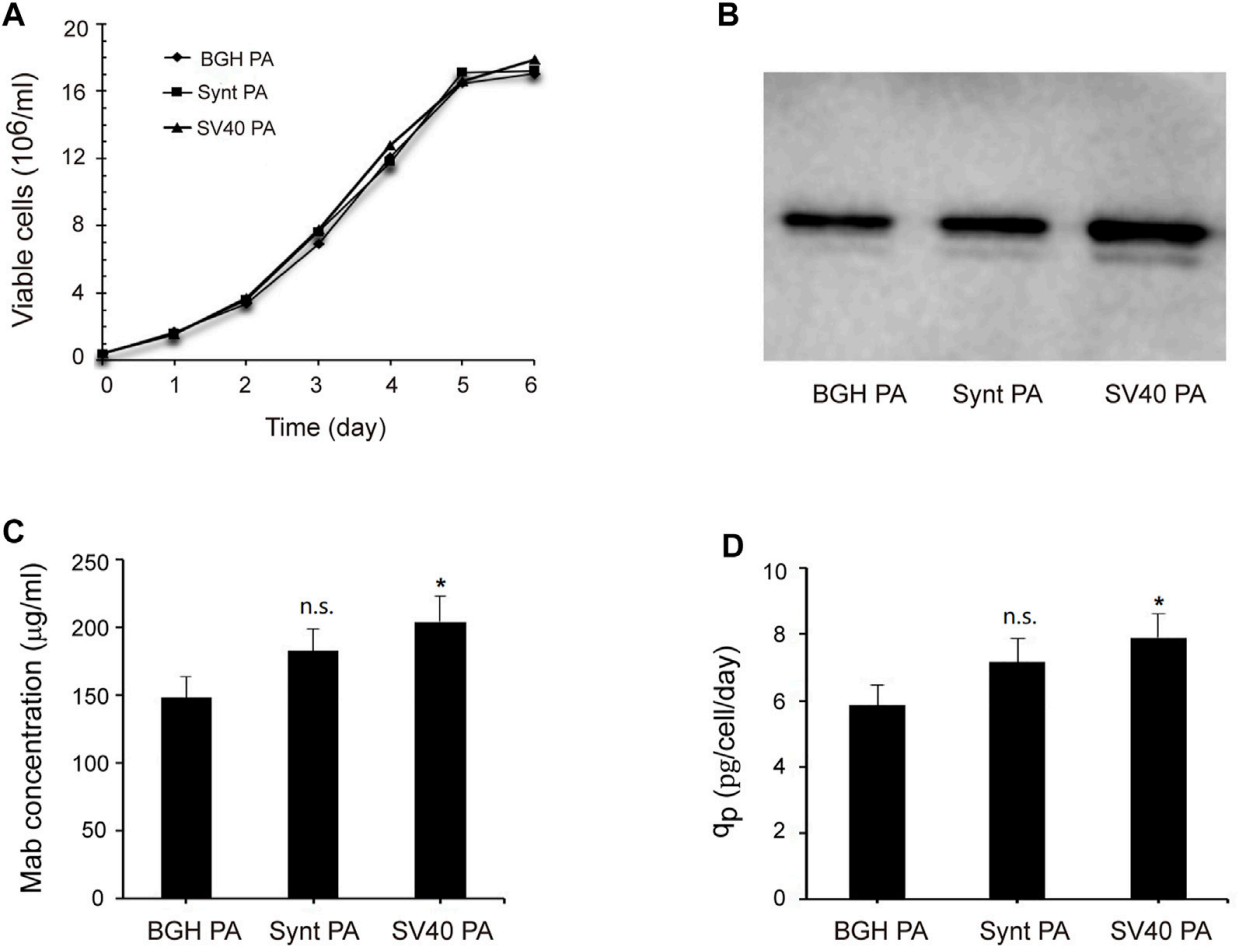
Analysis of adalimumab expression levels. The vectors containing adalimumab were transfected into CHO cells, and cells were selected with G418 to establish stable cell pools. Then, after 7 days of suspended culture, the supernatant was collected, and adalimumab expression was tested by Western blot and ELISA. **(A)** Viable cell concentration as a function of time; **(B)** Western blot results; **(C)**, mAb concentration; **(D)**, Cell-specific productivity (q_p_). The data were obtained from three independent experiments, n.s. *p* > 0.05, *p < 0.05.

To further investigate the effects of the Synt poly A and SV40 poly A on recombinant protein expression, we replaced the *eGFP r*eporter gene with the adalimumab [a monoclonal antibody (mAb)] sequence and analyzed its expression by Western blot and ELISA. Transfected cells were screened using G418 to generate the stable cell colonies and then cultured in 125-ml shake flasks in serum-free medium. After 7 days, the supernatants were collected, and adalimumab expression was analyzed (Figure 6). The results showed that the Synt poly A and SV40 poly A did not affect viable cell concentration (Figure 6A), and the adalimumab expression levels were consistent with eGFP expression, i.e., the vector with SV40 poly A and Synt poly A resulted in increasing mAb production (Figures 6B–D), compared with the BGH poly A, mAb concentration increased by 1.23- and 1.38-fold, q_p_ increased by 1.22- and 1.36-fold, respectively.

## Discussion

Expression vector optimization strategies have been highly effective for improving recombinant protein expression and stability in mammalian cells (Bayat et al., 2018; Yang et al., 2019; Li et al., 2020). The transgene expression is related to the type of cell line, the components of the vectors (Lee CP et al., 2018; Azzoni et al., 2007). In the present study, we investigated the effects of different poly A elements, including BGH, M BGH, Synt, HSV-TK, and SV40 poly A, on transgene expression and variation of expression in transfected CHO cells. The results indicated that polyadenylation sequences can influence transgene expression, different poly A showed different transgene expression levels, and the SV40 and Synt poly A significantly increased transgene expression levels and stability, decreased variation of expression.

pIRES-neo vector (Gene bank: U89673) containing CMV promoter and BGH poly A, was a common vector for mammalian system expression. In the present study, we used pIRES-neo as the original vector to systematically evaluate the effects of different poly A in transfected CHO cells. In the transient expression analyses, M BGH, Synt, HSV-TK, and SV40 poly A did not increase eGFP expression compared to BGH poly A, and GFP transient expression levels of M BGH PA and HSV TK PA was lower than others, which is consistent with the previous report (Azzoni et al., 2007). Previous studies have focused mainly on the impact of polyadenylation signals on cleavage-polyadenylation efficiency, plasmid nuclease resistance, and mRNA maturation/stability, and transgenes have only been investigated transient expression (Levitt et al., 1989; Wu and Alwine, 2004; Azzoni et al., 2007). In the current study, the transfected CHO cells were further cultured under low concentration G418 screening pressure, and eGFP expression was assessed at day 30 and day 120 post-transfection for stable and long-term stable expression, respectively. We observed that the SV40 poly A and Synt poly A elements positively affected transgene expression in stable transfected CHO cells, with eGFP stable expression and long-term stability expression appearing to be significantly enhanced. Compared to BGH poly A, SV40 poly A and Synt poly A enhanced stable and long-term stable eGFP expression levels by approximately 1.37- and 1.24-fold (stable), and 1.45- and 1.32-fold (long-term stable), respectively.

The transgene expression level is related to many factors, such as cell type, vector composition, the resistance of plasmid DNA to intracellular degradation, the levels of mRNA et al. The role and function of ployA at the post-transcriptional level are responsible for the differences in transient and stable transgene expressions. In this study, the BGH poly A (225 bp) was used as the control, whose pre-mRNA forms an extensive hairpin loop secondary structure at the 3′-untranslated region (Gimmi et al., 1989). The M BGH PA has a deletion of 61 bp downstream of the AATAAA signal, the deleted sequence included part of the 3′-efficiency elements of the BGH poly A (Ribeiro et al., 2004). SV40 poly A also forms a malleable stem-loop; however, the much smaller SV40 poly A signal has no GT or T-rich sequences upstream of the AATAAA sequence (Hans and Alwine 2000., Wu and Alwine 2004). The smallest HSV TK PA signal only 19 bp, in some extent, the longer and more malleable structure of the poly A signal may result in a more efficient polyadenylation sequence (Azzoni et al., 2007), this may cause a much lower GFP expression level compared with that of BGH poly A. Besides, the HSV TK PA yielded the lowest mRNA expression levels among the five poly A elements, which was responsible for a decrease in transgene expression. On the other hand, SV40 and Synt PA are more resistant to intracellular degradation (Azzoni et al., 2007), and plasmids with SV40 and Synt PA presented similar transient expression and higher stable expression to that obtained with plasmids BGH PA. Taken together the results indicate that the transgene expression level results from a compromise between plasmid nuclease resistance and post transcriptional efficiency of the poly A signal.

More importantly, the Synt and SV40 poly A elements were able to decrease the variation of transgene expression in stable transfected cells. At present, transgenic silencing and instability, and clonal heterogeneity can cause variations among stable transfected cell clones in CHO expression system (Lee JS et al., 2018; Lee et al., 2019). A considerable number of cell clones must be screened in order to obtain a highly productive and stable clone; a process which is time-consuming, laborious, and expensive. In this study, we observed that the Synt poly A and SV40 poly A sequences decreased the variation of transgene expression levels, which could significantly simplify the long and complicated procedure of generating stable high productive expression cell clones. Thus, the choice of the poly A element should be taken into consideration in the design of recombinant protein expression vectors.

To determine whether mRNA levels and gene copy numbers were responsible for influencing transgene expression in stable transfected cells, we performed qPCR analyses. Correlation analysis showed that long-term stable transgene expression might not be related to the gene copy number and mRNA level. In this study, SV40 poly A and Synt poly A increased transgene expression level, but no increase in the copy number and mRNA expression was found. These results indicate that the role of the SV40 poly A and Synt poly A elements in enhancing stable transgene expression is independent of gene copy number and mRNA level, and may be related to nuclease resistance and post-transcriptional regulation.

Finally, we evaluated the expression of the secreted protein, adalimumab, as mediated by the SV40 poly A and Synt poly A elements under serum-free medium culture conditions. Consistent with the previous studies (Harraghy et al., 2011; Xu et al., 2018), the expression levels of transgene were consistent with those of the eGFP reporter gene. Adalimumab expression levels in CHO cells transfected with plasmids containing SV40 poly A or Synt poly A sequences were significantly higher than those of cells transfected with the control plasmid containing the BGH poly A.

In this study, we first systematically investigated the effect of five poly A elements on transgene expression in transfected CHO cells and demonstrated the superiority of SV40 poly A and Synt poly A for stable and long-term stable expression, as well as reduced variation of expression of recombination proteins. Our study suggests that polyadenylation sequences play a crucial role in recombination protein expression, and our results are relevant for vector optimization in the production of recombinant proteins in stable transfected mammalian cells. SV40 poly A and Synt poly A were screened to be stronger elements that increase stable transgene expression and decrease the variation of expression, but only five poly A elements were selected and the number needs to be further expanded. In addition, the assembly of different elements on the vector also affected the transgenic expression, and the combination of poly A and other elements should be considered to achieve the ideal expression level of recombinant protein.

## Data Availability

The original contributions presented in the study are included in the article/Supplementary Material, further inquiries can be directed to the corresponding author.
