# Supplementary material for: Enhanced Transgene Expression by Optimization of Poly A in Transfected CHO Cells
Source: Front Bioeng Biotechnol. 2022 Jan 24;10:722722. doi: 10.3389/fbioe.2022.722722 (PMC8819543; doi:10.3389/fbioe.2022.722722)
Supplement: Supplementary file 2 [file Table2.DOCX]

**Figure S1 The sequence of ploy A elements and adalimumab**

**BGH polyA**

CTGTGCCTTCTAGTTGCCAGCCATCTGTTGTTTGCCCCTCCCCCGTGCCTTCCTTGACCCTGGAAGGTGCCACTCCCACTGTCCTTTCCTAATAAAATGAGGAAATTGCATCGCATTGTCTGAGTAGGTGTCATTCTATTCTGGGGGGTGGGGTGGGGCAGGACAGCAAGGGGGAGGATTGGGAAGACAATAGCAGGCATGCTGGGGATGCGGTGGGCTCTATGG

**Mutation BGH polyA**

CTGTGCCTTCTAGTTGCCAGCCATCTGTTGTTTGCCCCTCCCCCGTGCCTTCCTTGACCCTGGAAGGTGCCACTCCCACTGTCCTTTCCTAATAAAATGAGGAAATTGCATCGCATTGTCTGAAGACGGTGTCAGGCATGCTGGGGATGCGGTGGGCTCTATGG

**SV40 late polyadenylation signal**

CAGACATGATAAGATACATTGATGAGTTTGGACAAACCACAACTAGAATGCAGTGAAAAAAATGCTTTATTTGTGAAATTTGTGATGCTATTGCTTTATTTGTAACCATTATAAGCTGCAATAAACAAGTTAACAACAACAATTGCATTCATTTTATGTTTCAGGTTCAGGGGGAGGTGTGGGAGGTTTTTTAAAGCAAGTAAAACCTCTACAAATGTGGTA

**Synthetic polyA**

AATAAAAGATCTTTATTTTCATTAGATCTGTGTGTTGGTTTTTTGTGTG

**HSV TK polyA**

AATAAAAAGACAGAATAAA

**Adalimumab heavy chain** GAAGTGCAATTGGTTGAATCAGGAGGAGGACTCGTGCAACCGGGAAGGAGTTTACGATTATCTTGTGCTGCCTCTGGATTCACCTTTGACGACTATGCAATGCATTGGGTCCGTCAAGCACCAGGAAAAGGTTTAGAGTGGGTTTCAGCAATCACTTGGAACTCCGGACATATTGACTATGCCGATAGTGTTGAGGGTCGATTCACAATCTCACGAGATAACGCGAAGAATAGTCTATACCTACAGATGAATAGCCTAAGAGCTGAGGATACTGCCGTTTATTACTGTGCAAAGGTTTCCTATCTTTCTACTGCATCTAGTCTTGATTACTGGGGACAAGGAACACTTGTCACAGTTTCCTCTGCTAGCACAAAAGGACCTAGCGTTTTCCCTCTGGCACCATCAAGTAAGAGCACCAGTGGCGGGACAGCAGCACTGGGTTGTCTTGTGAAAGACTATTTCCCAGAACCCGTTACCGTTAGTTGGAACTCAGGCGCACTTACTTCGGGAGTTCATACTTTTCCTGCTGTCTTACAATCTTCCGGTCTCTATTCACTAAGCTCAGTTGTCACTGTACCTTCCTCAAGCCTTGGGACACAAACCTACATTTGTAACGTCAATCATAAACCGAGCAATACGAAGGTAGATAAGAAAGTCGAGCCAAAGAGTTGTGATAAAACACACACTTGCCCACCTTGCCCAGCTCCTGAACTCTTAGGTGGACCAAGCGTTTTCCTCTTTCCTCCAAAGCCGAAAGATACACTTATGATATCACGCACACCCGAAGTTACTTGTGTGGTTGTAGACGTTTCTCATGAAGATCCCGAAGTGAAGTTTAATTGGTACGTCGATGGTGTTGAAGTTCACAATGCTAAGACTAAGCCAAGAGAAGAGCAATACAACTCAACCTATAGAGTTGTTTCCGTCTTAACCGTACTGCATCAAGATTGGTTGAACGGCAAGGAGTATAAATGCAAGGTTAGCAATAAAGCACTACCTGCACCGATTGAGAAGACAATTAGCAAAGCAAAAGGACAACCAAGGGAACCACAAGTCTATACACTTCCACCTTCAAGGGATGAGCTGACTAAGAATCAAGTATCCTTGACCTGTTTAGTCAAGGGGTTTTACCCTTCTGACATTGCCGTAGAATGGGAATCTAATGGGCAGCCTGAGAATAACTATAAGACAACTCCACCCGTACTCGATTCTGACGGCTCTTTTTTCCTATACTCCAAGCTAACCGTGGATAAATCACGTTGGCAACAAGGAAACGTTTTCTCTTGTTCTGTGATGCACGAGGCTTTGCATAATCACTACACACAAAAGAGCTTAAGTCTTAGCCCTGGGAAATAG

**Adalimumab light chain** GACATACAAATGACTCAATCTCCAAGTTCACTATCTGCTTCAGTCGGCGATAGGGTCACTATAACTTGTAGAGCCTCTCAGGGCATAAGAAACTATTTGGCATGGTACCAACAGAAACCTGGAAAAGCTCCTAAGCTGCTAATATATGCTGCTTCTACACTTCAGAGTGGAGTACCTTCAAGATTCAGTGGATCTGGTTCTGGGACTGATTTCACTTTGACTATCTCATCCCTCCAACCAGAAGACGTTGCTACATACTATTGCCAGCGCTATAATAGGGCTCCTTATACCTTTGGACAAGGCACAAAAGTCGAGATTAAGAGAACTGTTGCTGCACCATCAGTGTTTATTTTCCCTCCAAGTGACGAACAGCTTAAATCTGGAACTGCAAGCGTTGTATGCCTTCTCAACAATTTCTACCCTAGAGAAGCGAAAGTCCAATGGAAAGTAGATAACGCACTTCAGTCTGGGAACTCACAAGAGAGTGTCACTGAACAAGATTCGAAAGACTCTACCTATTCACTCTCATCGACTCTTACTCTGTCAAAAGCTGATTACGAGAAGCACAAAGTGTATGCTTGCGAAGTTACACACCAAGGACTTAGCTCACCAGTAACCAAGAGCTTCAATAGGGGAGAATGCTGA
